# Shear force comparative evaluation for surface treated and non- treated 3D interim printed materials with different types of glass-ionomer cements

**DOI:** 10.4317/jced.57003

**Published:** 2020-10-01

**Authors:** Anna-Maria Latz, Constantin von See, Vasilios Alevizakos, Maximilian Sandmair, Ahmed Othman

**Affiliations:** 1 Center of digital dentistry and CAD/CAM technology - Danube Private University- Krems- Austria; 2 Prof. Dr. Center of digital dentistry and CAD/CAM technology - Danube Private University- Krems- Austria; 3 Dr. med. Dent. Center of digital dentistry and CAD/CAM technology - Danube Private University- Krems- Austria; 4 Dr. Center of digital dentistry and CAD/CAM technology - Danube Private University- Krems- Austria

## Abstract

**Background:**

The purpose of this investigation was to compare shear force of different glass-ionomer cements on 3D printed interim material in combination with and without surface pretreatment.

**Material and Methods:**

120 rectangular specimens made of printable provisional material (Bego, Bremen, Germany) were used. After post-processing the specimens were blasted with aluminum oxide 110µm (Bego, Bremen, Germany). Extra 120 non-surface treated specimens were used as an experimental negative test group. All 240 specimens were divided randomly into 6 groups. All were cemented with a compressive load of 20 N using universal testing machine Z010 (Zwick/Roell, Ulm, Germany) to ensure a comparable cementing process. Each of the six groups were cemented with different cements (CX Plus (Shofu, Ratingen, Germany), Vivaglass CEM PL (Ivoclar Vivadent AG, Schaan, Liechtenstein), Aqua Cem (Dentsply Sirona, Bensheim, Germany), Ketac Cem (3M, Neuss, Germany), Meron Plus AC (Voco, Cuxhaven, Germany), and Fuji 1 (GC, Tokyo, Japan). Shear force test was performed, and forces were statistically analyzed via Anova test (significance level *p*<0.001).

**Results:**

All the pre-treated specimens showed a significantly higher bonding strength compared to not pretreated. Meron Plus AC showed the highest shear overall force. The Anova test showed a significant difference between all pretreated study groups (*p*<0.001).

**Conclusions:**

An increase of the necessary forces for all groups was shown in pretreated group. Within the limitations of this study, a surface pretreatment is recommended when bonding a 3D interim material with glass ionomer cements.

** Key words:**Shear force, 3D printing, glass ionomer cement, mechanical evaluation, CAD/CAM.

## Introduction

During the last decade, the use of computer-aided design and manufacturing (CAD/CAM) in dentistry has been increasing, which was triggered by progress in intra-oral scanning and manufacturing technologies (1-5). Research into and production of materials suiTable for CAD/CAM processing is one of the fastest-growing and -changing fields in dental materials (6). As shown in industry, high precision, simpler fabrication protocol and minimal human intervention are the benefits of computerized engineering technology (7). These advantages make CAD/CAM ideal for quality assurance, precision production and cost-effective manufacturing (1,8). Under these circumstances, it is not surprising that the CAD/CAM technology has been implemented in dentistry more and more (2,9). Today, the CAD/CAM-approach is known for durable tooth-colored and metal-free components in dental practice, providing chair-side fabrication of indirect restorations.

For luting these restorations several materials are available nowadays. One of these materials is the glass ionomer luting cement. The glass ionomer luting cement was introduced to the dental field as it has a wide range of potential applications. A higher force can be achieved by surface treatment or even some adhesive systems depending on the materials used (10).

The chemically adhesive ingredients interact with the tooth surfaces and prosthodontic materials, allowing bonding of fixed partial denture prosthesis and the tooth. In fact, this is leading to a reinforcement of the tooth and its restoration (11).

As 3D printing resin for provisional crowns and bridges has been recently developed, there is only limited data available on the bonding strength of various luting materials and different surface pre-treatments (8,12).

The aim of this study was to compare different glass-ionomer cements using shear force tests on a 3D printed interim restoration material in combination with and without surface treatment.

## Material and Methods

-Material

The specimens in the present study were digitally designed (Autodesk Netfabb, San Rafael, CA, USA), 3D printed (Varseo S, Bego, Bremen, Germany) and post-processed (Otoflash, Bego, Bremen, Germany) in line with the manufacturer’s instructions. The specimens were made of prinTable resin for temporary restorations (VarseoSmile Temp A2, Bego, Bremen, Germany). An unheated ultrasonic reusable ethanol jar with a concentration of 96% was used to clean the specimens for 3 minutes followed by 2 more minutes of a new ethanol bath with 96% concentration. The specimens were withdrawn from the ethanol bath and dried with compressed air. The surface polymerization was performed using Otoflash (Bego, Bremen, Germany) including Nitrogen gas (1.0-1.2 bar) flashing with a frequency of 10 Hz. After a period of 1500 flashes the samples were turned around for another 1500 flashes.

Overall a number of 240 specimens were investigated.

One half had a rectangular shape (n=120) and the other half a cylindrical shape (n=120).

120 specimens were pretreated by blasting their surface with aluminum oxide (110µm).

The non-surface treated specimens (n=120) were used as a negative test.

As luting material six different glass ionomer cements were used.

- Fuji I (GC, Tokyo, Japan)

- Vivaglass CEM PL (Ivoclar Vivadent, Schaan, Liechtenstein)

- CX-Plus (Shofu, Ratingen, Germany)

- AquaCem (Dentsply Sirona, Bensheim, Germany)

- Meron Plus AC (Voco, Cuxhaven, Germany)

- Ketac Cem (3M, St. Paul, Minnesota, USA)

**Figure 1:**
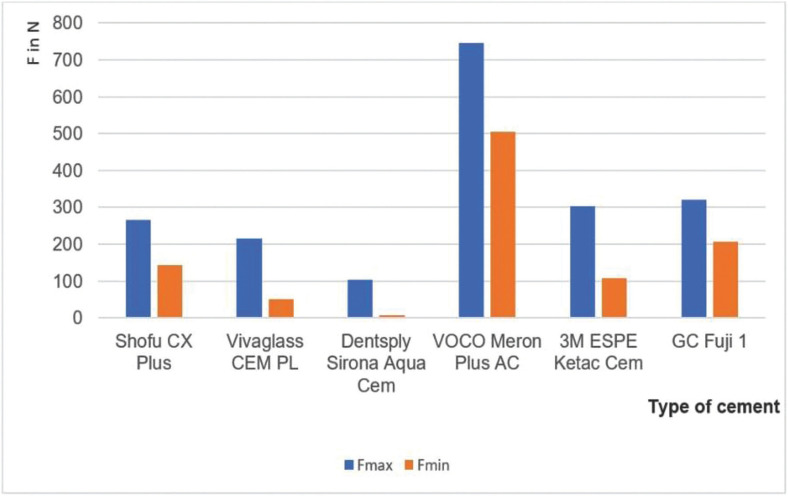
Maximum and minimum shear force of each cement.

**Figure 2:**
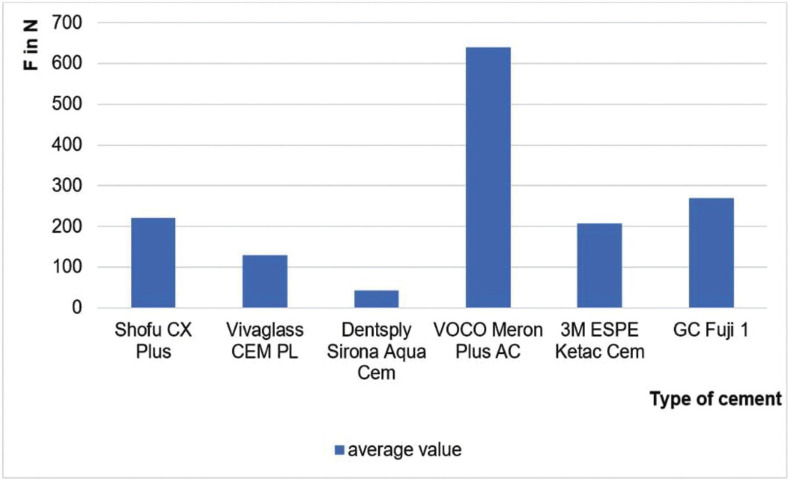
Average shear force of each cement.

**Figure 3:**
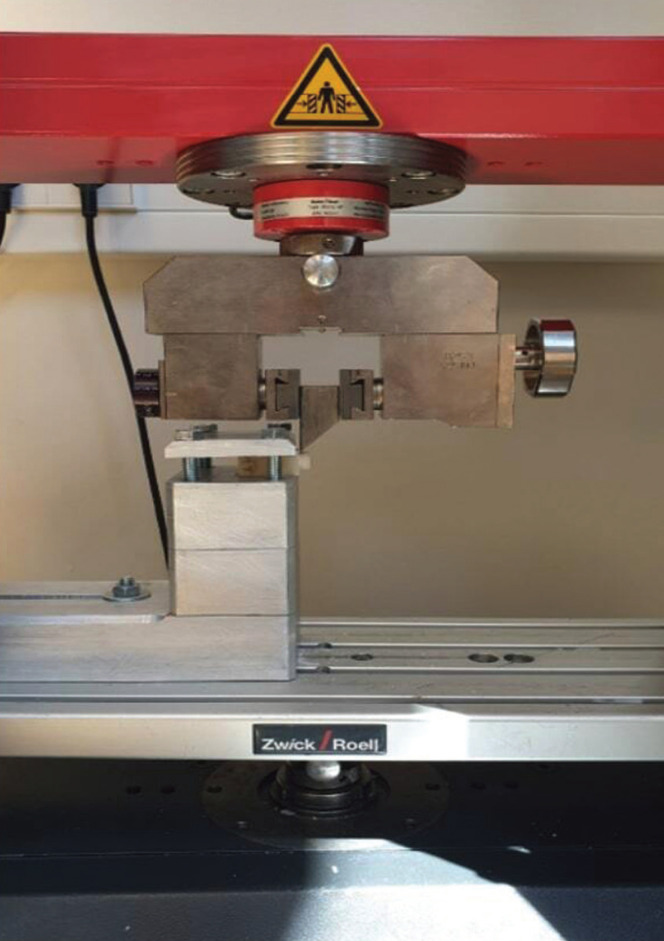
Setup in the universal testing machine Z010.

Apart from Meron Plus AC and GC Fuji I, having an automix dispenser, all the other cements had to be mixed manually, (Figs. 1-3).

-Methods

The pretreated (n=120) and non-surface treated (n=120) rectangular and cylindric specimens were randomly pair wisely divided into six experimental groups. The groups were associated with one glass ionomer luting cement used for bonding in each group. The surface treated specimens were divided in 60 cylindric and 60 rectangular shapes.

About 100 mg of mixed cement was applied on the rectangular and cylindrical specimens bonding surfaces. The rectangular and cylindrical pair specimens were then immediately loaded by the universal testing machine Z010 (Zwick/Roell, Ulm, Germany), and excess material was removed with microbrushes (Micro applicator brush, Ultradent products, South Jordan, United States) directly after loading and before complete chemical curing. After applying the luting cements, a load of 20 N had been held for 6 minutes. The cement line wasn’t modified, and no protective varnish was applied. The finished test samples were kept dry, with the exclusion of daylight. After about 24 hours, the shear force testing according to the ISO 11405/2003 (0.75 +/- 0.3 mm/min) was started using the universal testing machine.

The adhesive surface of the test specimens was loaded with a chisel made of hardened steel exerting force on the specimen pairs directly next to the cement connection. The crosshead moved until the cement connections broke. All results were recorded in Newton (N). The maximum values for each specimen tested were recorded and specified as shear force.

-Statistical analysis

The recorded values were statistically analyzed performing a K-S-Test. All measurements can be considered as normally distributed. An ANOVA test was performed with a highly significant difference between the groups (*p*<0.05) using SPSS V24.0 Software (IBM, Armonk, USA).

## Results

The six groups were statistically analyzed using the Kolmogorov-Smirnov Adaptation Test (K-S-test) (Table 1). All groups could be considered as normally distributed (*p*> 0.001).

**Table 1:**
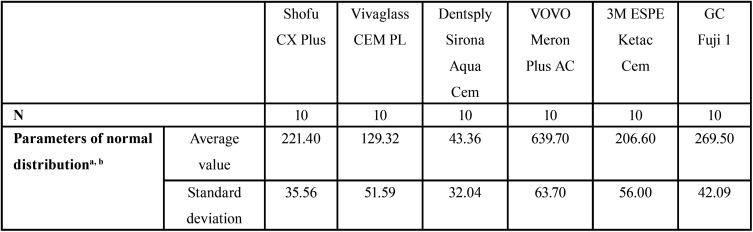
The statistical analysis using the Kolmogorov-Smirnov Adaptation Test (K-S-test).

For the negative control groups (not pretreated) the data must be interpreted with cautions as they are in the minimal range of the machines testing settings (Table 2).

**Table 2:**
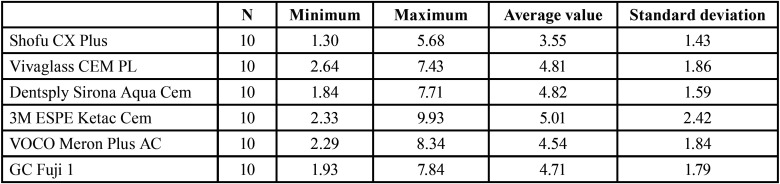
The descriptive analysis of all groups (non surface-treated) regarding the withstanded loading force [N].

As seen in this Table the study should only be conducted with prior treatment. There are no significant results without surface treatment found within this study. These are the reasons why the first control group was not further investigated.

The highest overall loading force of 747 N was reached with Meron Plus AC and pretreatment while the lowest load force with pretreatment of 103 N was reached with Aqua Cem (Table 3). The average force of all cements is also illustrated in Table 3.

**Table 3:**
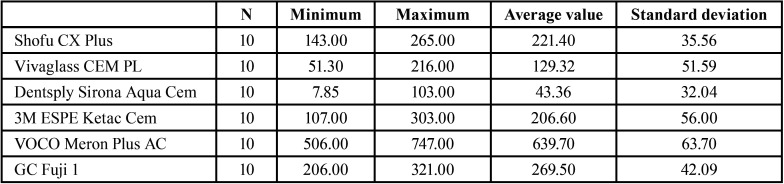
The descriptive analysis of all groups (pretreated) regarding the withstanded loading force [N].

To estimate the level of significance, the Anova test was applied. This resulted in a high significance (*p*>0.001) (Table 4).

**Table 4:**
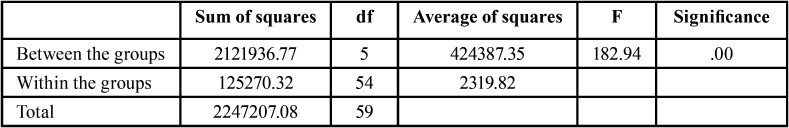
The Anova test was applied. This resulted in a high significance (*p*>0.001).

Meron Plus AC on the pretreated surface showed with a force of 747 ± 63.7 N a significantly higher shear force than all other groups.

## Discussion

The aim of the present study was to investigate the maximum shear force of different glass ionomer luting cements on pretreated surfaces of 3D printed blocks made of printable resin for provisional restorations.

It was shown that Meron Plus AC withstood a significantly higher force to VarseoSmile Temp than all other tested cements.

According to several studies in literature, investigating the shear force of glass ionomer cement, the surface of all specimens was manually pretreated by blasting (13,14). In the present study, the surface was blasted by aluminum oxide (110µm) from 1cm distance in an angulation of 45° for the investigation group. Possible deviations in the distance or angle during the blasting process might possibly result in differences in the surface. However, studies have shown that blasted surfaces enable significantly higher forces than non-surface treated surfaces (13,15). The printing supports were positioned on the non-cementing surfaces. The universal testing machine was used for reproducible loaded forces during the cementation process on all specimens (n=240) for 6 minutes (16). Microbrushes were used immediately after machine loading to eliminate excess cement to prevent adulteration of the specimen’s adhesive surfaces.

In the present investigation, significant differences between the used glass ionomer cements were found. These results are in line with Blixt *et al.* who found higher force of surface pre-treatment with aluminum oxide (17). Furthermore, the data revealed that the shear force of the six tested glass ionomer cement groups had different maximum and minimum values. A possible reason for that could be the different chemical components of the tested glass ionomer cements and their interaction with the VarseoSmile Temp material. Furthermore, the size of the incorporated fillers in the different luting materials is unknown. Fillers with bigger diameters than the used aluminum oxide particles will not be able to jam on the surface created after blasting. In literature, it is shown that the filler size is the most important factor in penetrating into the surface after conditioning to obtain high force (18). Also, the viscosity has to be taken into account. Less viscous material causes better wetting and results in a bonded surface with fewer defects and higher force (19). The mixing method also has an impact on material properties, especially when mixed manually. Incorrect mixing may negatively affect mechanical properties (20).

As a limitation of this study, glass ionomer luting material and chemically cured cements were used only. Additionally, it was only possible to test a limited number of samples and no long-term observations were made. These restrictions were made because this study was focused on short-term impacts and this study is a pilot study. Further investigations in this field, as well as surface modifications after mechanical pretreatment, are necessary to better understand these complex relationships.

In literature, different cement adhesion values can be found with resin cements having higher values than resin modified glass ionomer cements (21). A study conducted by Piwowarczyk *et al.* showed higher shear bond strength for resin cements than resin modified glass ionomer cements on zirconia ceramic material (22).

Comparing the shear force values of different glass ionomer resin cements used in this study to those of other similar protocols is difficult because every investigation is performed with different operator specifications. Accordingly, the presented data and values for force could only be compared inside the same study. In general, these study results are near to the study conducted by Peutzfeldt *et al.* (23). The material resistance for further clinical consideration should be more widely investigated. The assumed value of 20 MPa for secure fixation could not be refuted nor proven to these days (24). Some more detailed studies are required, especially the chemical polymerization process within the 3D printable resin needs further investigation. The main factor which might influence the results was the shrinkage of the cement during polymerization which might cause stress on the composite layer. However, the effects of these parameters and possible interactions with VarseoSmile Temp should be analyzed in future studies as several aspects need further research.

## Conclusions

Within the limitations of the present study, it could be concluded that Meron Plus AC reaches higher shear forces compared to other cements. In addition, a clear increase of force for all groups was shown when the surface was pretreated with aluminum oxide. Further studies are needed to investigate the micro-retentive junction and the influence of different filler sizes on the shear force.

## References

[1] van Noort R (2012). The future of dental devices is digital. Dent Mater.

[2] Miyazaki T, Hotta Y (2011). CAD/CAM systems available for the fabrication of crown and bridge restorations. Aust Dent J.

[3] Rekow ED (2006). Dental CAD/CAM systems: a 20-year success story. J Am Dent Assoc.

[4] Beuer F, Schweiger J, Edelhoff D (2008). Digital dentistry: an overview of recent developments for CAD/CAM generated restorations. Br Dent J.

[5] Miyazaki T, Hotta Y, Kunii J, Kuriyama S, Tamaki Y (2009). A review of dental CAD/CAM: current status and future perspectives from 20 years of experience. Dent Mater J.

[6] Ruse ND, Sadoun MJ (2014). Resin-composite blocks for dental CAD/CAM applications. Journal of dental research.

[7] Abduo J, Lyons K (2013). Rationale for the use of CAD/CAM technology in implant prosthodontics. International journal of dentistry.

[8] Jeong KW, Kim SH (2019). Influence of surface treatments and repair materials on the shear bond strength of CAD/CAM provisional restorations. J Adv Prosthodont.

[9] Kapos T, Ashy LM, Gallucci GO, Weber HP, Wismeijer D (2009). Computer-aided design and computer-assisted manufacturing in prosthetic implant dentistry. Int J Oral Maxillofac Implants.

[10] Mazaheri R, Pishevar L, Shichani AV, Geravandi S (2015). Effect of different cavity conditioners on microleakage of glass ionomer cement with a high viscosity in primary teeth. Dental research journal.

[11] Rohr N, Fische J (2017). Tooth surface treatment strategies for adhesive cementation. J Adv Prosthodont.

[12] Holmer L, Othma A, Lührs AK, von See C (2019). Comparison of the shear bond strength of 3D printed temporary bridges materials, on different types of resin cements and surface treatment. J Clin Exp Dent.

[13] Śmielak B, Klimek L (2018). Effect of Air Abrasion on the Number of Particles Embedded in Zironia. Materials (Basel).

[14] Shankar R, Tripathi A, Singh RD, Chand P (2010). Adhesion of different brands of glass ionomer cements to a ceramometal alloy. Journal of Indian Prosthodontic Society.

[15] Ramashanker Singh RD, Chand P, Jurel SK, Tripathi S (2011). Evaluation of adhesive and compressive strength of glass ionomer cements. Journal of Indian Prosthodontic Society.

[16] Chen B, Yang L, Lu Z, Meng H, Wu X, Chen C (2019). Shear bond strength of zirconia to resin: The effects of specimen preparation and loading procedure. J Adv Prosthodont.

[17] Blixt M, Adamczak E, Lindén LA, Odén A, Arvidson K (2000). Bonding to densely sintered alumina surfaces: effect of sandblasting and silica coating on shear bond strength of luting cements. Int J Prosthodont.

[18] Najafi-Abrandabadi A, Najafi-Abrandabadi S, Ghasemi A, Kotick PG (2014). Microshear bond strength of composite resins to enamel and porcelain substrates utilizing unfilled versus filled resins. Dental research journal.

[19] Bing H, Dong Y, Gao X, Wang X, Tian F (2012). Effect of filler content on the microtensile bond strength of composite resin and dentin in Class I cavities. Quintessence International.

[20] Sulaiman TA, Abdulmajeed AA, Altitinchi A, Ahmed SN, Donovan TE (2018). Effect of Resin-modified Glass Ionomer Cement Dispensing/Mixing Methods on Mechanical Properties. Oper Dent.

[21] Abad-Coronel C, Naranjo B, Valdiviezo P (2019). Adhesive Systems Used in Indirect Restorations Cementation: Review of the Literature. Dent J (Basel).

[22] Piwowarczyk A, Lauer HC, Sorensen JA (2005). The shear bond strength between luting cements and zirconia ceramics after two pre-treatments. Oper Dent.

[23] Peutzfeldt A, Sahafi A, Flury S (2011). Dentin bonding of cements. The bonding of cements with dentin in combination with various indirect restorative materials. Schweiz Monatsschr Zahnmed.

[24] Bayne SC (2012). Correlation of clinical performance with 'in vitro tests' of restorative dental materials that use polymer-based matrices. Dent Mater.

